# Fungi of entomopathogenic potential in *Chytridiomycota* and *Blastocladiomycota*, and in fungal allies of the *Oomycota* and *Microsporidia*

**DOI:** 10.1186/s43008-021-00074-y

**Published:** 2021-10-11

**Authors:** Agata Kaczmarek, Mieczysława I. Boguś

**Affiliations:** 1 Witold Stefański Institute of Parasitology, Polish Academy of Sciences, Twarda 51/55, 00-818 Warsaw, Poland; 2 Biomibo, Strzygłowska 15, 04-872 Warsaw, Poland

**Keywords:** Biological control, Host-specificity, Insect pathogens

## Abstract

The relationship between entomopathogenic fungi and their insect hosts is a classic example of the co-evolutionary arms race between pathogen and target host. The present review describes the entomopathogenic potential of *Chytridiomycota* and *Blastocladiomycota* fungi, and two groups of fungal allies: *Oomycota* and *Microsporidia*. The *Oomycota* (water moulds) are considered as a model biological control agent of mosquito larvae. Due to their shared ecological and morphological similarities, they had long been considered a part of the fungal kingdom; however, phylogenetic studies have since placed this group within the *Straminipila*. The *Microsporidia* are parasites of economically-important insects, including grasshoppers, lady beetles, bumblebees, colorado potato beetles and honeybees. They have been found to display some fungal characteristics, and phylogenetic studies suggest that they are related to fungi, either as a basal branch or sister group. The *Blastocladiomycota* and *Chytridiomycota*, named the *lower fungi*, historically were described together; however, molecular phylogenetic and ultrastructural research has classified them in their own phylum. They are considered parasites of ants, and of the larval stages of black flies, mosquitoes and scale insects.

## INTRODUCTION

The interaction between host and parasite is characterised on the one hand by the parasites developing more effective strategies of host exploitation, and on the other, by the hosts mounting increasingly robust defences though Red Queen dynamics or coevolutionary arms races. Furthermore, depending on gene flow and differences in selection pressure between sites, both host and parasite may demonstrate local adaptation to their counterpart or develop more general resistance or offensive traits (Kaur et al. 2019).

Although chemical pesticides have been used for the control of insect pests for many decades, they are known to exert side effects on non-target organisms, contaminate groundwater and leave residues on food crops; they are also believed to support the development of insect resistance. Therefore, there is currently great interest in the development of alternative methods for integrated pest management (Kim et al. 2017; Mužinić and Želježić 2018; Neuwirthová et al. 2019). More recent studies have found entomopathogens to regulate many populations of arthropods (Lacey et al. 2001; Lacey et al. 2015; Lacey 2016), and that entomopathogenic fungi themselves may play a role in removing harmful substances and heavy metals from the environment (Litwin et al. 2020).

The success of using an entomopathogenic fungus as a myco-biocontrol agent depends on the selection of a virulent and stable strain with specific efficacy for the target host. Hence, there has been considerable interest in the creation of new strains of entomopathogenic fungi, accompanied by the development of fermentation systems for optimal biomass production, and the design of delivery systems suitable for both the microorganism and common agricultural practices. One key step in the development of effective strains involves the cloning of genes that can enhance pathogenesis (St. Leger and Wang 2010; Jaronski and Mascarin 2017; Dobrowolski et al. 2018; Karabörklü et al. 2018).

The co-evolution of fungi and insects over hundreds of millions of years has resulted in the development of a wide range of complex and intricate interactions between them (Joop and Vilcinskas 2016). Entomopathogenic fungi have evolved to infect a wide range of insects in all developmental stages (viz. eggs, larvae, pupae, nymphs and imago) across a range of niches. Such adaptability requires considerable morphological diversity, resulting in a wide range of fungal species with morphological, phylogenetic and ecological diversity. However, although recent advances in the genome biology of entomopathogenic fungi indicate the genetic bases of their adaptation to insect hosts and host ranges, as well as the evolutionary relationships between insect and non-insect pathogens (Araújo and Hughes 2016; Wang and Wang 2017), the mechanisms of host specificity in pathogenic microbiology generally remain poorly understood.

## EVOLUTION OF ENTOMOPATHOGENIC FUNGI

From an evolutionary point of view, entomopathogenic fungi do not constitute a monophyletic group. Instead, phylogenetic data suggests that entomopathogenic activity has arisen independently, and frequently, along the course of fungal evolution (Humber 2008; Araújo and Hughes 2016; Moonjely et al. 2016). Wang and Wang (Wang and Wang 2017) proposed that this evolutionary pattern is indicative of frequent cross-kingdom or cross-phylum host jumping during fungal pathogen speciation. Such phenomena may be explained by the *host-habitat hypothesis* or the *host-relatedness hypothesis*. The host-habitat hypothesis suggests that entomopathogens accidently switch between host organisms living in a common environment; this better explains the dynamic evolution of host diversification and adaptation in the Hypocreales (Nikoh and Fukatsu 2000; Spatafora et al. 2007; Blackwell 2010). In contrast, the host-relatedness hypothesis suggests that pathogens change hosts to new species closely related with the original host (Nikoh and Fukatsu 2000; Kepler et al. 2012).

Historically, the *Fungi* were divided into four phyla; however, following dramatic changes in higher-level taxonomy in the last 20 years, the number of fungal phyla has tripled to 12, organized into six major groups: *Dikarya, Mucoromycota, Zoopagomycota, Blastocladiomycota, Chytridiomyceta* and *Opisthosporidia* (James et al. 2020). It has been estimated that about 750 species of entomopathogenic fungi exist, most of which are distributed in the phyla *Chytridiomycota, Blastocladiomycota, Zoopagomycota*, *Basidiomycota* and *Ascomycota*. Some authors have also traditionally included the *Microsporidia* and the ecologically-similar, but phylogenetically-distinct, *Oomycota* (water moulds), belonging to the *Straminipila*.

## *OOMYCOTA*

The *Oomycota* are a genetically and morphologically-diverse clade that can form hyphae or exist as simple holocarpic thalli; the phylum contains at least 1500 species of fungus in 100 genera (Beakes and Thines 2017). The oomycetes are filamentous eukaryotic microorganisms belonging to the kingdom *Straminipila*; however, some authors placed them among the fungi, based on their ecological and morphological characteristics, viz. filamentous growth habit, nutrition by absorption, and reproduction via spores (Rossman and Palm 2006; Beakes et al. 2012; Thines 2018). Unlike the true fungi, the cell walls of the oomycetes are composed of cellulose derivatives that serve as structural components, rather than chitin (Hassett et al. 2019; Klinter et al. 2019). Sexual reproduction typically occurs between gametangia (antheridia and oogonia) on the same or different hyphae (Rocha et al. 2018; Spring et al. 2018); however, this is not always the case: *Periplasma isogametum*, for example, demonstrates a type of sexual reproduction involving morphological isogamy and physiological anisogamy (Martin and Warren 2020). Most oomycetes are also capable of asexual reproduction via their kidney bean-shaped biflagellate zoospores with apically or laterally-attached whiplash and tinsel flagella (heterokonts) (Walker and van West 2007). Interestingly, the mitochondria of the oomycetes possess tubular cristae, as opposed to the disc-like cristae of the fungi (Powell et al. 1985; Karlovsky and Fartmann 1992; Weber et al. 1998; Rossman and Palm 2006).

Pathogenic oomycetes are able to infect a broad range of algae, plants, protists, fungi, arthropods and vertebrates, including humans, and can cause losses in agriculture and aquaculture (Mendoza and Vilela 2013; van West and Beakes 2014; Kamoun et al. 2015). Twelve species of entomopathogenic *Oomycota* are known to exist within six genera: *Lagenidium* (*L. giganteum*), *Leptolegnia* (*L. caudata* and *L, chapmanii*), *Pythium* (*P. carolinianum*, *P. sierrensis,* and *P. flevoense*), *Crypticola* (*C. clavulifera* and *C. entomophaga*), *Couchia* (*C. amphora, C. linnophila*, and *C. circumplexa*), and *Aphanomyces* (*A. laevis*) (Araújo and Hughes 2016; Mendoza et al. 2018). Most are facultative parasites of mosquito larvae belonging to the genera *Anopheles, Culex, Aedes, Ochlerotatus, Culiseta, Orthopodomyia, Uranotaenia, Psorophora,* and *Mansonia* (Frances et al. 1989; Woodring et al. 1995; Scholte et al. 2004; Patwardhan et al. 2005; Vyas et al. 2007; Singh and Prakash 2012; Vilela et al. 2018; Shen et al. 2019; Vilela et al. 2019; Shen et al. 2020a). Some examples of infected insects are listed in Table 1.

**Table 1:** Examples of insects infected by entomopathogenic fungi and fungal-like organisms

	Infected insect species	Literature data
***Oomycota***		
*Aphanomyces laevis*	Mosquito larvae	(Patwardhan et al. 2005)
*Couchia* spp*.*		(Wallace Martin 2000)
*Crypticola* spp*.*		(Frances 1991; Dick 2003; Mendoza et al. 2018)
*Lagenidium giganteum*		(Golkar et al. 1993; Vyas et al. 2007; Vilela et al. 2019)
*Leptolegnia caudata* spp.		(Bisht et al. 1996; Montalva et al. 2016; Gutierrez et al. 2017)
*Pythium* spp.		(Clark et al. 1966; Su et al. 2001; Su 2008; Shen et al. 2019)
***Microsporidia***		
*Anncaliia algerae*	*Drosophila melanogaster*	(Sokolova et al. 2020)
*Antonospora locustae (Nosema locustae)*	Grasshoppers	(Lange 2005; Zhou and Zhang 2009; Gerus et al. 2020)
*Nosema adaliae*	Two-spotted lady beetle, *Adalia bipunctata* (*Coleoptera*: *Coccinellidae*)	(Steele and Bjørnson 2014)
*Nosema alticae*	Flea beetle, *Altica hampei* (Coleoptera: Chrysomelidae)	(Yıldırım and Bekircan 2020)
*Nosema bombi, Nosema ceranae, Tubulinosema pampeana*	Bumblebees	(Brown 2017)
*Nosema carpocapsae*	Codling moth, *Cydia pomonella* L	(Zimmermann et al. 2013)
*Nosema leptinotarsae*	Colorado potato beetle, *Leptinotarsa decemlineata* Say. (Coleoptera: Chrysomelidae).	(Bekircan 2020)
*Nosema maddoxi*	Brown marmorated stink bug*, Halyomorpha halys* (Hemiptera: Pentatomidae)	(Preston et al. 2020b; Preston et al. 2020a)
*Nosema pernyi*	The Chinese oak silkworm *Antheraea pernyi* (Lepidoptera: Saturniidae)	(Liu et al. 2020)
*Nosema pyrausta*	European corn borer *Ostrinia nubilalis,* the Asian corn borer *Ostrinia furnacalis* and the adzuki bean borer *Ostrinia scapulalis*	(Grushevaya et al. 2018; Grushevaya et al. 2020)
*TubuliNosema* sp.	*Loxostege sticticalis L (*Lepidoptera: Crambidae*)*	(Malysh et al. 2018)
*TubuliNosema suzukii*	*Drosophila suzukii*	(Biganski et al. 2020)
*Vairimorpha (Nosema) ceranae Vairimorpha (Nosema) apis*	Honeybee *Apis mellifera*	(Forsgren and Fries 2010; Tokarev et al. 2018; Applegate and Petritz 2020; Chang et al. 2020; Ostroverkhova et al. 2020)
***Chytridiomycota***		
*Myrmicinosporidium durum*	Ants from Subfamilies: Myrmicinae, Formicinae and Dolichoderinae	(Sanchez-Peña et al. 1993; Gonçalves et al. 2012; Trigos-Peral et al. 2017)
*Nephridiophaga maderae Nephridiophaga archimandrita* *Nephridiophaga lucihormetica* *Nephridiophaga blattellae* *Nephridiophaga blaberi*	Madeira cockroach (*Leucophaea maderae*), *Archimandrita tessellate* *Lucihormetica verrucose* German cockroach *Blattella germanica* Death’s head cockroach *Blaberus craniifer*	(Radek et al. 2017) (Radek et al. 2011) (Radek and Herth 1999) (Fabel et al. 2000)
***Blastocladiomycota***		
*Coelomycidium simulii*	Larval stage of black flies (Diptera): *Simulium asakoae, S. chamlongi, S. chiangmaiense, S. fenestratum, S. feuerborni, S. nakhonense, S. nodosum, S. quinquestriatum, S. tani,* and *S. japonicum*	(Levchenko et al. 1974; Kim 2011; Jitklang et al. 2012; Kim 2015; Adler and McCreadie 2019)
*Coelomomyces africanus* *Coelomomyces angolensis* *Coelomomyces iliensis* *Coelomomyces indicus* *Coelomomyces irani* *Coelomomyces lairdi* *Coelomomyces maclaeyae* *Coelomomyces numularius* *Coelomomyces opifex* *Coelomomyces pentangulatus* *Coelomomyces polynesiensis* *Coelomomyces punctatus* *Coelomomyces solomonis* *Coelomomyces santabrancae* spp.	Mosquito larvae from species of *Culex, Culiseta, Aedes, Anopheles, Psorophora,* and *Uranotaenia*	(Scholte et al. 2004; Balaraman et al. 2006; Rueda-Páramo et al. 2017; Dahmana and Mediannikov 2020; Gao et al. 2020)
*Myiophagus* cf. *ucrainicus*	Scale insect: Caribbean black scale, *Saissetia neglecta* (Hemiptera), Caliphornia red scale, *Aonidiella aurantii* (Hemiptera) *Chironomus atracinus* larvae	(Moore and Duncan 2017) (Czeczuga and Godlewska 2001)

The most well-known and thoroughly-analysed entomopathogenic *Oomycota* is arguably *L. giganteum*, which was used as a biological control model of mosquito larvae (Vilela et al. 2019). Infection is initiated by free-floating zoospores formed in sporangia, which are released only in aqueous environments (Walker and van West 2007). The spores are responsible for recognising and binding to the host cuticle. After successful binding, they swell, germinate and penetrate the insect exoskeleton. The mycelia grow through the hemocoel, resulting in the death of the insect, while the fungus terminates with reproduction and the subsequent release of infectious zoospores. Successful infection requires the production of hydrolytic enzymes such as chitinases (Shen et al. 2020b), hexosaminidases (Dowd et al. 2007; Olivera et al. 2016) or glycoside hydrolase family 5 subfamily 27 (GH5_27), which play crucial roles in the cuticle-degrading process (Quiroz Velasquez et al. 2014). Other important enzymes produced by entomopathogenic oomycetes are trehalases, which not only take part in cuticle degradation, but may also hasten the infection process by depleting trehalose, the most abundant sugar source in insect hemolymph (McInnis and Domnas 1973).

It is possible that the ancestors of *L. giganteum* were plant pathogens, as sequence analyses have indicated the presence of genes characteristic of plant tissue infections, such as *crn* or *cbel*. However, other genes coding cuticle-degrading enzymes have been found, such as GH5_27 or GH20; these are characteristic of entomopathogens and are not observed in plant pathogens (Quiroz Velasquez et al. 2014; Olivera et al. 2016). This dichotomy suggests that fungal and oomycete entomopathogens not only share morphology and pathological strategies, but also evolutionary histories and ecological relationships (Leoro-Garzon et al. 2019; Shen et al. 2019).

*L. giganteum* was used as a control agent against mosquito larvae in the commercial product Laginex, which was registered and released in the USA in 1995 (Hallmon et al. 2000); however, due to several cases of life-threating mycoses in dogs recorded in the southern United States, the product was later deregistered and is no longer for sale (Mendoza and Vilela 2013; Vilela et al. 2015; Vilela et al. 2019). Recent data has shown the existence of two forms of *L. giganteum* (Vilela et al. 2015): the heat-tolerant taxon *L. giganteum* f. *caninum* pathogenic to mammals (Spies et al. 2016)*,* and *L. giganteum f. giganteum,* which can infect mosquito larvae in nature. Both taxa share a common ancestor and can infect mosquito larvae; the only phenotypic difference between the two types is their tolerance for growth at different temperatures: the latter does not tolerate human body temperature (37°C) but develops well at 25°C and below (Vilela et al. 2015; Vilela et al. 2019).

Oomycetes have also been found to infect non-dipteran insects; for example *Crypticola entomophaga* has been observed to attack aquatic insects from the order *Trichoptera* (caddis flies; Dick 2003; Gani et al. 2019).

## *MICROSPORIDIA*

*Microsporidia* are unicellular eukaryotes living as obligate intracellular parasites. All stages of their life-cycle associated with growing and replication can only take place inside host cells; in the environment, they can survive only as thick-walled spores (Timofeev et al. 2020). Their adaptation to a parasitic strategy has resulted in the development of a seemingly paradoxical mixture of characteristics: the cells lack mitochondria, and their metabolism does not employ electron transfer chains, oxidative phosphorylation, or the tricarboxylic acid (TCA) cycle. In addition, their genomes are poor in genes involved in resource-producing metabolic pathways, such as ATP synthesis, but rich in others that enhance transport mechanisms and allow resources to be hijacked from the host. Due to their highly-reduced morphology, ultrastructure, biochemistry and metabolism, as well as their considerably impoverished genome, microsporidia need to induce considerable disruption of host cell physiology to enable successful infection and development (Corradi 2015; Haag et al. 2019; Timofeev et al. 2020). One exception to this rule is *Microsporidium daphniae*, which has been found to possess a mitochondrial genome and the genes necessary for producing ATP from glucose (Haag et al. 2014).

Of all known parasites, those of *Microsporidia* are arguably the most host dependent (Corradi and Keeling 2009; Keeling 2009; Tamim El Jarkass and Reinke 2020), and this dependence may account for their development of a range of mechanisms to ensure intracellular survival. For example, they demonstrate reduced expression of immune-peptide or immune-related genes (Antúnez et al. 2009), reduced re-epithelization in infected ventriculi (Higes et al. 2007; García-Palencia et al. 2010), increased energetic stress (Mayack and Naug 2009; Martín-Hernández et al. 2011) and inhibited melanisation in the hemolymph of infected *Bombyx mori* larvae (Wu et al. 2016).

The life-cycle of microsporidia is characterized by three phases: an infective or environmental phase (spores), a merogony (proliferative) phase and a sporogonic or spore-forming phase (Keeling and Fast 2002). Microsporidia spores are first ingested or inhaled by the host. The spores then germinate and produce a long and convoluted tubule extrusion apparatus (polar tubule); this tubule distinguishes them from other organisms and plays a crucial role in host cell invasion. The spore approaches the host cell, and its polar tubule is everted to enter the cell and inject its sporoplasm into the host cell cytoplasm. Following successful injection, the proliferative phase begins; this phase includes all cell growth and division from the sporoplasm until spore formation. It is known to include two possible processes: schizogony and merogony. Although very little is understood of schizogony in *Oomycota*, merogony is known to take place as the injected sporoplasm develops into meronts (the proliferative stage); these in turn multiply by binary fission or multiple fission, depending on the species, to form multinucleate plasmodial forms. Finally, these forms undergo sporogony, with the meronts developing into sporonts, which produce two or more sporoblasts; these in turn undergo metamorphosis into spores.

Interestingly, unlike other species, in which sporogony occurs in the presence of plasmalemmal thickening, the microsporidia undergo sporogony to produce diplokaryotic nuclei after meiosis. These *sporonts* can multiply by binary or multiple fission, acquire specialized organelles and become spores. Subsequently, the spores continue the cycle by spreading through the tissues of the host, infecting new cells (Lallo et al. 2016; Han and Weiss 2017; Han et al. 2020; Horta et al. 2020).

Traditionally, *Microsporidia* were classified within the phylum *Apicomplexa* as *sporozoan parasites*; however, phylogenetic studies suggest that microsporidia are related to fungi, either as a basal branch or sister group (Lee et al. 2008; Han and Weiss 2017). They have also been found to include chitin in the spore wall and use trehalose as a sugar reserve, and studies have noted the presence of closed mitosis, spindle pole bodies and meiosis (Han and Weiss 2017). Although they can infect a great number of domestic and wild animals, the most common hosts are arthropods and fish. Since their discovery in the 1850s as the causative agent of the silkworm disease pebrine, or *pepper disease*, which devastated the silk industry in Europe, these pathogens have demonstrated major economic significance in animal farming, such as nosemosis in beekeeping (*Nosema aApis* and *N. ceranae*) and microsporidiosis in aquaculture (*Loma salmonae* for salmonids and *Thelohania* spp. for shrimp); *Nosema bombycis* has also been found to infect the domesticated silkworm *Bombyx mori.* In addition, some microsporidia act as opportunistic pathogens of humans, especially in patients with AIDS (Weiss 2020).

About 93 genera of microsporidia have the ability to infect insects (Becnel and Andreadis 2014). They are considered as biological control agents for regulating the population of the beet webworm *Loxostege sticticalis,* responsible for serious damage to crops such as soybean, sugar beet, alfalfa and sunflower in northern Eurasia (Malysh et al. 2020). Interestingly, some authors consider microsporidia to have enabled the invasive success of the ladybird *Harmonia axyridis* (Vilcinskas et al. 2015; Verheggen et al. 2017)*,* in which the microsporidia act as a symbiotic “biological weapon” against some predators, like *Coccinella septempunctata* and *Adalia bipunctata* (Ceryngier et al. 2018). Some examples of infected insects are listed in Table 1.

Also, *Antonospora locustae*, formerly known as *Nosema locustae* (syn. *Paranosema locustae*), is commonly used as a biological control agent for grasshoppers in the commercial products Nolo Bait and Semaspore (Lange 2005; Zhou and Zhang 2009; Solter et al. 2012). Like other microsporidial pathogens, *A. locustae* also needs to control the metabolic processes and molecular programmes of the host in order to proliferate. Successful infection was found to suppress the locust gut microbiota, including the gut bacteria that produce aggregation pheromones, thus preventing host aggregation (Pan et al. 2018).

Most microsporidia species require two successive host generations to complete their life-cycle, and at least three genera, viz. *Amblyospora*, *Hyalinocysta* and *Parathelohania*, also require development in an intermediate copepod host (Becnel and Andreadis 2014). These genera are also highly host- and tissue-specific, with complex developmental sequences comprising unique stages and events (Becnel et al. 2005). They are mostly pathogens of mosquito larvae (Andreadis 1985; Andreadis 2007; Shen et al. 2020a); interestingly, microsporidian infection has also been found to be associated with a reduction in *Plasmodium falciparum* transmission in *Anopheles arabiensis* mosquitoes (Herren et al. 2020).

In contrast, some species of microsporidia exhibit simple life-cycles with a single spore type responsible for oral (horizontal) transmission; these affect only one generation of insects and are not usually host or tissue specific (Becnel et al. 2005). A good example of a genus with this simple life-cycle is *Nosema*, including the species *N. apis* and *N. ceranae.* These species are responsible for most microsporidian infections in bees and other species of *Hymenoptera*, resulting in ecological and economical losses in apiculture (Higes et al. 2006; Forsgren and Fries 2010; Grupe and Alisha Quandt 2020; Ostroverkhova et al. 2020; Paudel et al. 2020). Interestingly, phylogenetic studies have placed the *Nosema* species able to infect bees within a new genus, *Vairimorpha* (Tokarev et al. 2020)*.*

*N. apis* is believed to have long been a parasite of the European honeybee *Apis mellifera*, whereas *N. ceranae* is presumed to have more recently undergone a host shift to *A. mellifera* from the Asian honeybee *A. ceranae* (Buczek et al. 2020; Ostroverkhova et al. 2020). The parasites exhibit a tissue tropism for honey bee ventriculus (Higes et al. 2020) and are believed to infect their ventricular epithelial cells during digestion; infected bees suffer impaired nutrient absorption, resulting in energy loss and death by starvation (Valizadeh et al. 2020).

*Nosema pernyi*, a microsporidium infecting the Chinese oak silkworm *Antheraea pernyi*, can enter the gut cell by polar tube infection in most situations. The spore in the polar tube germinates within the alkaline environment of the intestine, inducing destructive chronic disease (Wang et al. 2019; Han et al. 2020). It is also possible for the parasite to exploit the metabolism of the host cell to progress its own life-cycle. A recent study found that successful infection of the honeybee midgut by the microsporidian pathogen *N. ceranae* involves the inhibition of apoptosis (Higes et al. 2013; Kurze et al. 2018); in such cases, the parasite appears to inhibit apoptosis by regulating the genes involved in the process, as well as in the cell cycle (Martín-Hernández et al. 2017), thus increasing oxidative stress and antioxidant enzyme generation in the gut, and inhibiting the genes involved in the homeostasis and renewal of intestinal tissues (Dussaubat et al. 2012; Liu et al. 2020).

Microsporidial infection can also alter host metabolism and induce both local and systemic innate immune responses (Pan et al. 2018). For example, *N. ceranae* infection is believed to suppress immune defence mechanisms in honey bees; studies have indicated that infection downregulates some immune-related genes, including *abaecin, apidecin, defensin, hymenoptaecin, glucose dehydrogenase (GLD)* and *vitellogenin (Vg)*, and upregulates Jun-related antigen *Jra* and *pi3k* (Chang et al. 2020). Additionally, transcription of the inhibitor of apoptosis protein (*iap-2*) gene was found to be upregulated in *Nosema*-tolerant honeybees (Kurze et al. 2015); it is possible that inhibition of apoptosis might help the parasites optimize their environment and extend the period during which they can grow and differentiate within host cells (Higes et al. 2013). It also appears that pheromone production in worker and queen honeybees to be modified during infection (Dussaubat et al. 2013).

## *CHYTRIDIOMYCOTA*

The *Chytridiomycota* comprise zoosporic fungi phylogenetically related to the true fungi. This group comprises at least three major lineages of chytrids: (1) *Rozella* spp., the earliest diverging lineage in kingdom *Fungi*; (2) *Olpidium brassicae,* the only species classified in *Zygomycota*;*,* and (3) the *core chytrid clade*, encompassing the remaining orders and families and most flagellated fungi, including those of *Chytridiales, Spizellomycetales* p.p., Monoblepharidales and *Neocallimastigales* (Barr 2001; James et al. 2006). Chytrids are unicellular, colonial or filamentous organisms with absorptive nutrition, and which reproduce through the production of motile zoospores, typically propelled by a single, posteriorly-directed flagellum (Barr 2001).

Infection begins with the attachment of a motile zoospore to the surface of a host cell, and the formation of a thickened wall around the zoospore. In a successful infection, the encysted zoospore will develop into a mature sporangium, following which, a germ tube typically forms, which enters the host cell via the girdle zone. The zoospores form within the host cell and are released to infect other cells by dehiscence. The attached zoospores completely depend on the host cell for nourishment and their development into mature sporangia (Ibelings et al. 2004).

Traditionally, *Chytridiomycota* have been regarded as aquatic fungi (Ibelings et al. 2004), but most species occur in soil as saprophytes growing on organic material (Digby et al. 2010). In addition, certain obligate anaerobic species also occur in the digestive tract of herbivores, and these are probably very important in the digestion of cellulose and hemicellulose (Li and Heath 1993; Paul et al. 2018; Saye et al. 2021). A few halophytes have been found in estuaries (Powell et al. 2015). A significant number of species are known as parasites of algae, vascular plants, amphibians, rotifers, tardigrades, protists (Luttrell 1974; Barr 2001; Ibelings et al. 2004) and invertebrates such as nematodes (Betancourt-Román et al. 2016) and insects (Sinha et al. 2016). The first described parasites of vertebrates were isolated from frog skin (Longcore et al. 1999).

The chytrids include one entomopathogenic genus: the monotypic *Myrmicinosporidium* (*M. durum*). That species is an obligate endoparasite of various ant hosts with a global or wide-ranging specific distribution (Lapeva-Gjonova 2014; Trigos-Peral et al. 2017)*.* Infected ants can be distinguished by their darker colour and larger abdomen size. Although the numbers of lentiform spores within an infected ant may initially be very low, they increase during infection, to the point where they can be found throughout the whole body, numbering more than one hundred in a single ant (Sanchez-Peña et al. 1993; Pereira 2004; Espadaler and Santamaria 2012; Gonçalves et al. 2012). Although the spores develop extensively in the ant haemocoel, an infected ant does not exhibit significant differences in life span or behaviour from uninfected ones (Sanchez-Peña et al. 1993); however, several studies have reported the early death of infected ants after hibernation (Espadaler and Santamaria 2012; Giehr and Heinze, 2015). Some examples infected of insects are listed in Table 1.

Recent studies have also placed the nephridiophagids in *Chytridiomycota* (Strassert et al. 2020). The systematic position of the nephridiophagids has been discussed intensively, and they were historically placed with the haplosporidians or microsporidians (Radek et al. 2017). A preliminary molecular analysis placed them within the *Fungi*, close to the *Zygomycota* (Wylezich C, Radek R 2004); however, a later phylogenetic analysis of SSU and LSU rRNA gene sequences placed them in the phylum *Chytridiomycota* (Strassert et al. 2020).

Nephridiophagids are unicellular, spore-forming parasites which infect the Malpighian tubules of insects, especially cockroaches (*Dictyoptera*) and beetles (*Coleoptera*), where they are mainly found in the lumen (Radek et al. 2011). The life-cycle of the nephridiophagids includes a merogony phase with vegetative multinucleate plasmodia that divide into oligonucleate and uninucleate cells. The sporogonial plasmodia form internal, 5–10 μm long, oval, flattened spores, generally with one nucleus, with the residual nuclei of the mother cell remaining in the cytoplasm between them (Radek et al. 2017).

## *BLASTOCLADIOMYCOTA*

Historically, the fungi belonging to the phylum *Blastocladiomycota* were described together with the *Chytridiomycota*; however, recent molecular phylogenetic and ultrastructural research has classified them in their own phylum (James et al. 2006; Porter et al. 2011). Genome-scale trees suggest *Blastocladiomycota* may have diverged around the same time as *Chytridiomycota* (James et al. 2020).

The *Blastocladiomycota* contains only the order *Blastocladiales*; this group contains zoospore-producing true fungi, with well-developed hyphae, closed mitosis, cell walls with β-1–3-glucan, and a secretory vesicle complex known as the Spitzenkörper (James et al. 2020; Roberson 2020). Several of these were once model organisms (e.g., *Allomyces* and *Blastocladiella*; (Cantino et al. 1968; Burke et al. 1972), and obligate parasites of plants and animals (Longcore and Simmons 2012). They are divided into five families (Barr 2001): *Blastocladiaceae* which contains only saprobic species; *Catenariaceae* with both saprobes and pathogens; *Coelomomycetaceae* with pathogens of invertebrates; *Physodermataceae* with obligate parasites of plants; and *Sorochytriaceae* which contains a pathogen infecting tardigrades. Powell (2017) subsequently described a sixth family, *Paraphysodermataceae*, which includes parasites of algae.

The entomopathogenic *Blastocladiomycota* are found in the genera *Coelomomyces* and *Coelomycidium* of *Coelomomycetaceae*. These genera include approximately 70 species pathogenic to insects such as mosquitoes and flies (Powell 2017; Shen et al. 2020a). Some examples of insects infected by *Blastocladiomycota* are listed in Table 1.

*Coelomycidium simulii* is a widespread species pathogenic to black flies, being mostly found in the larvae, and rarely in pupae and adults. Infected larvae have large numbers of minute, spherical thalli throughout the body cavity. These thalli give rise to spores that are released into the water column after the death of the host. An intermediate host might be required to complete the life-cycle (McCreadie and Adler 1999; Boucias and Pendland 2012).

The species of *Coelomomyces* are aquatic fungi that act as obligate parasites. They are best known as pathogens of mosquito larvae; however, some might infect aquatic dipteran insects including those of the *Psychodidae*, *Chironomidae*, *Simuliidae* and *Tabanidae* (Scholte et al. 2004; Shen et al. 2020a). Although they are probably not host specific, they nevertheless have relatively restricted host ranges (Federici 1981). The life-cycle of *Coelomomyces* is complex and comprises obligatory development in an intermediate microcrustacean host (cyclopoid copepods, harpacticoid copepods, or ostracods) and two mosquito generations. Infection is also known to cause significant epizootics which can persist in larval populations for several years, resulting in mortality rates greater than 50%, and often higher than 90% (Scholte et al. 2004). Moreover, the fungus might remain inside the insect, passing through the larval and pupal stages to mature in the ovaries of adult females, where the sporangia start to produce zoospores. After the first blood meal, these zoospore-filled sporangia are ‘laid’ by the female mosquito, ready to infect new larvae, instead of eggs (Araújo and Hughes 2016). Due to the relatively specific host range of the fungus, being generally restricted to mosquitoes, and the devastating effects of natural epizootics on larval populations, it has been proposed as a suitable biocontrol of mosquito populations. However, these advantages have to be weighed against its unpredictable infection rate, complicated life-cycle and problem with mass production (Scholte et al. 2004).

The fungi placed in *Myiophagus* were initially considered to belong to *Chytridiomycota* (Blackwell and Powell 2020); however, based on their morphological differences, they are now placed in *Blastocladiomycota* (Humber 2012; James et al. 2014; Powell 2017). Species of this genus infect leeches (Czeczuga et al. 2003), scale insects, mealybugs, beetle larvae, and dipteran pupae (Karling 1948) resulting in chytridiosis in subtropical regions (Tanada and Kaya 2012). As *Myiophagus* requires free water for dissemination (Cole 2012), infection takes place shortly after or during heavy rainfall. In such conditions, motile zoospores are released from resting sporangia and these swim through the water film that forms over “drip leaves” to find potential hosts; similar to other aquatic fungi, the zoospores are believed to be guided to the host by specific chemoreceptors (Luisa 2012).

## CONCLUSION

Biological control is an effective and environmentally-acceptable alternative to chemical insect control methods. Entomopathogenic fungi and their metabolites are believed to represent potential alternatives to chemical pesticides. When used as biocontrol agents, entomopathogens might offer several advantages over conventional insecticides, such as high efficiency and selectivity, safety for beneficial organisms, reduction of residues in the environment, and increased biodiversity in human-managed ecosystems. However, relatively little is known of their effectiveness in field applications and the potential side effects of their use. It is also important to emphasise that due to the global distribution and variety of insects, many as yet unknown entomopathogenic fungi may exist; as such, there is a clear need for further comprehensive studies of the biology and ecology of entomopathogenic organisms and of the mechanisms underlying their action on insect hosts.

## Data Availability

Not applicable.
